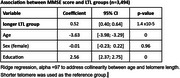# Associations between telomere length and cognitive performance in a long‐lived elderly population from Costa Rica: findings from the CRELES Study

**DOI:** 10.1002/alz70855_099717

**Published:** 2025-12-23

**Authors:** Carolina Ochoa‐Rosales

**Affiliations:** ^1^ Latin American Institute for Brain Health (BrainLat), Universidad Adolfo Ibañez, Santiago, Metropolitan Region, Chile

## Abstract

**Background:**

The ends of human chromosomes are protected by a structure of DNA and proteins containing repeating TTAGGG sequences, known as telomeres. Telomere length serves as a proxy of biological age. Longer telomeres are linked to longer healthspan, while shorter telomeres are associated with age‐related diseases such as dementia, regardless of chronological age. However, this has not been extensively studied in the Latin American population. We investigated associations between leukocyte telomere length (LTL) and cognitive performance in long‐lived elderly individuals from Costa Rica.

**Method:**

We included *n* = 3,494 participants from the Costa Rican Longevity and Healthy Aging Study (CRELES), a nationally representative survey of health and life course experiences of Costa Ricans aged ≥60 years. A short version of the Mini‐Mental State Examination (MMSE) test (up to 15 points), was used to collect data on cognitive performance. MMSE score ≤12 indicated cognitive impairment (CI). LTL was assayed using quantitative polymerase chain reaction. After calculating LTL in base‐pairs, participants were categorized into short telomeres (lowest quartile of length in base‐pairs) or long telomeres (≥quartile 2). We explored associations between MMSE score (dependent variable) and LTL across short and long telomeres groups, using multivariable linear regression adjusted for age, sex and education as covariates. We introduced Ridge regularization to account for multicollinearity between LTL and age.

**Result:**

The median age was 62 years (IQR:11), with a maximum age of 110 years. Women constituted 58.0% of the sample, 74.6% had only primary education or no formal education, and 59.0% had CI. Across LTL categories, 57.2% of individuals in the long telomeres group and 64.6% in the short telomeres group had CI. We found a significant association between MMSE score and LTL, wherein individuals in the long telomere group had, on average, 0.52‐points higher MMSE score (coefficient=0.52, 95%CI 0.40‐0.64, *p*‐value <0.0001), compared with the short telomere group, independent of confounders (Table 1).

**Conclusion:**

This is the first study in Latin America to suggest that better cognitive performance is cross‐sectionally associated with longer telomeres, independent of chronological age, sex and educational attainment. Longitudinal studies are needed to better assess the correlation between cognition and telomere attrition during aging.